# De-Doped Polyaniline as a Mediating Layer Promoting In-Situ Growth of Metal–Organic Frameworks on Cellulose Fiber and Enhancing Adsorptive-Photocatalytic Removal of Ciprofloxacin

**DOI:** 10.3390/polym13193298

**Published:** 2021-09-27

**Authors:** Xinyu Hou, Lijian Sun, Ying Hu, Xianhui An, Xueren Qian

**Affiliations:** Key Laboratory of Bio-Based Material Science & Technology, Northeast Forestry University, Ministry of Education, Harbin 150040, China; hxy@nefu.edu.cn (X.H.); lantian0308@nefu.edu.cn (L.S.); huying@nefu.edu.cn (Y.H.); anxianh509@163.com (X.A.)

**Keywords:** cellulose fiber, metal–organic frameworks, polyaniline, de-doping, adsorption, photocatalysis, ciprofloxacin

## Abstract

New kinds of inorganic–organic hybrid porous materials, metal–organic frameworks (MOFs), have shown great application potential in various fields, but their powdery nature limits their application to a certain extent. As a green and renewable biomass material in nature, cellulose fiber (CelF) has the advantages of biodegradability, recyclability and easy processing, and can be used as an excellent flexible substrate for MOFs. However, the efficient deposition of MOFs on CelF is still a great challenge for the development of this new material. Herein, polyaniline (PANI) and de-doped PANI (DPANI) with rich functional groups as a mediating layer was proposed to promote the in-situ growth and immobilization of some MOFs on CelF. The PANI (especially DPANI) layer greatly promoted the deposition of the four MOFs, and more encouragingly, significantly promoted the in-situ growth and nanocrystallization of MIL-100(Fe). MIL-100(Fe)@DPANI@CelF was selected as an adsorbent-photocatalyst to be used for the adsorptive-photocatalytic removal of ciprofloxacin (CIP) in water. The removal efficiency of CIP by MIL-100(Fe)@DPANI@CelF reached 82.78%, and the removal capacity of CIP was as high as 105.96 mg g^−1^. The study found that DPANI had a synergistic effect on both the in-situ growth of MIL-100(Fe) on CelF and the adsorption-photocatalysis of CIP in water. The universal platform of PANI-mediated in-situ growth and immobilization of MOFs on CelF constructed in this study widens the road for the development of MOF@CelF composites.

## 1. Introduction

Metal–organic Frameworks (MOFs) are a class of crystalline materials that consist of coordination bonds between transition-metal cations and multidentate organic linkers [1], which have the advantages of both inorganic compounds and organic polymers. In recent years, MOFs and their composites have shown great application potential and value in many fields, such as conduction [2], antibacterial applications [3], gas storage [4], water purification [5], drug delivery [6], adsorption [7,8], photocatalysis [9], etc. However, the crystalline nature of MOFs determines their powder form, and their recycling and processability are affected to a certain extent, significantly limiting their application. As a green and renewable resource in nature, cellulose has the advantages of biodegradability, recyclability, high flexibility and easy processing [10,11,12], which make it particularly suitable as an excellent flexible carrier of MOFs. At the same time, the existence of MOFs will also give cellulose many new functions, which can be described as killing two birds with one stone.

Up to now, there has been positive progress in the research and development of cellulose fiber-based MOF (MOF@CelF) composites. Paper and pulp fiber [13], cotton fabric [14,15], nanocellulose [16,17], aerogel [18,19] and bacterial cellulose [20] have all been used as substrates for the loading of MOFs. However, the loading of MOFs on some untreated CelF (e.g., pulp fibers and cotton fibers) is generally low in amount and weak in binding affinity. Therefore, some studies were conducted to promote the loading of MOFs on CelF, to a certain extent, by means of carboxymethylation [21,22], atomic layer deposition [23], citric acid modification [24], (3-aminopropyl)triethoxysilane (APTES) and 3-glycidyloxypropyltrimethoxysilane (GPTMS) as connecting substances [25,26,27], polydopamine as an intermediate layer [20], etc. However, the degradation of CelF is inevitable during carboxymethylation, and atomic layer deposition has high requirements for equipment. At the same time, there are few studies on the efficient binding of MOFs with CelF. There is an urgent need to find an efficient and simple universal method to promote the in-situ growth and immobilization of MOFs on CelF on the basis of maintaining the inherent morphological characteristics of CelF.

As a common conductive polymer, polyaniline (PANI) has excellent chemical and thermal stability, and it shows strong adsorption on metal ions [28]. Meanwhile, its photocatalytic efficacy is also phenomenal [29]. ZIF-8, ZIF-67, HKUST-1 and MIL-100(Fe), as four MOFs that can be generated under green synthesis conditions at room temperature, are not only in line with the concept of sustainable development, but also have great potential for the development of emerging CelF-based composites.

Ciprofloxacin (CIP) is a widely used fluoroquinolone that is frequently detected in different natural water bodies [30]. Therefore, it is of great urgency to develop effective techniques to remove CIP from aqueous environments and mitigate its inherent risks to ecosystems [31]. CIP can be removed by means of physical adsorption [31], chemical oxidation [30] and photodegradation [32]. Among the four above-mentioned MOFs, MIL-100(Fe) is often used as a part of photocatalytic composites due to the existence of Fe^3+^ [33,34], and shows excellent removal ability for CIP in water; therefore, it plays a distinctive role in water environment treatment.

In this work, the promoting effect of PANI and de-doped PANI as a mediating layer on the in-situ growth and immobilization of the four MOFs (ZIF-8, ZIF-67, HKUST-1 and MIL-100(Fe)) on CelF (i.e., bleached softwood pulp) were investigated. Firstly, a thin PANI layer, with a large number of quinone nitrogen structures as a mediator, was formed in-situ on CelF to provide rich nucleation sites for the in-situ growth of MOFs. Then, the PANI@CelF was treated by ammonia to obtain the de-doped PANI@CelF (abbreviated as DPANI@CelF), and the above-mentioned four MOFs were further in-situ grown on the DPANI@CelF. MIL-100(Fe)@DPANI@CelF was used as a novel adsorptive photocatalyst to treat CIP wastewater. The effect of the MIL-100(Fe) deposition ratio and the DPANI layer on the synergistic adsorptive-photocatalytic removal of CIP was discussed.

## 2. Experiment

### 2.1. Materials and Reagents

Cellulose fiber (CelF, Canadian bleached kraft softwood pulp board) was kindly provided by Mudanjiang Hengfeng Paper Co., Ltd. (Mudanjiang, China), and beaten to 37° SR using a PL4-2 speed governing beater before use. Ferrous chloride tetrahydrate (≥99.7%) was produced by Tianjin Guangfu Technology Development Co., Ltd. (Tianjin, China), cobalt nitrate hexahydrate (AR, 99%) by Aladdin Biochemical Technology Co., Ltd. (Shanghai, China), zinc nitrate hexahydrate (AR, 99%) by Tianjin Damao Chemical Reagent Factory (Tianjin, China), copper acetate (≥99.0%) by Zhiyuan Chemical Reagent Co., Ltd. (Tianjin, China), 2-methylimidazole (2-MI, ≥98.0%) by Guangfu Fine Chemical Research Institute (Tianjin, China), trimesic acid (H_3_BTC, 98%) by Macklin Biochemical Co., Ltd. (Shanghai, China), sodium hydroxide (≥96.0%) by Tianda Chemical Reagent Factory (Tianjin, China), absolute ethanol (≥99.7%) by Yongda Chemical Reagent Co., Ltd. (Tianjin, China), hydrochloric acid (diluted to 0.6 M before use) and ammonia (diluted to 0.1 M before use) by Kermel Chemical Reagent Co., Ltd. (Tianjin, China), aniline (ANI, purified by vacuum distillation before use) and hydrogen peroxide (30%) by Tianli Chemical Reagent Co., Ltd. (Tianjin, China), and ammonium persulfate (APS, ≥98.0%) by Fuchen Chemical Reagent Co., Ltd. (Tianjin, China). Ciprofloxacin hydrochloride (CIP) was of USP grade (≥88.5%).

### 2.2. Preparation of PANI@CelF and DPANI@CelF

Typically, 2 g of CelF (oven dry basis), 180 mL of HCl (0.6 M) solution and 1 mL of ANI were added successiely to a 500 mL three-necked bottle in an ice-water bath (0–5 °C), and stirred for 40 min to ensure that the CelF dispersed evenly in the reaction medium. Then, 20 mL of HCl (0.6 M) solution containing 4.9 g APS was slowly dropped into the above system under stirring. After 105 min, the treated fibers were filtered and washed with tap water to obtain PANI@CelF.

The resultant PANI@CelF and 200 mL ammonia solution (0.1 M) were put into a 500 mL three-necked bottle and stirred for 1 h for de-doping treatment. The product was filtered and washed with tap water until the filtrate was colorless to obtain the de-doped PANI@CelF (abbreviated as DPANI@CelF).

### 2.3. Preparation of MOF Composites

The preparation of MOF composites is briefly described, taking MOFs@CelF composites as examples. For the preparation of MOFs@PANI@CelF and MOFs@DPANI@CelF, it was only necessary to replace CelF with PANI@CelF and DPANI@CelF. For comparison, powdery MOFs were also prepared without adding CelF.

#### 2.3.1. Preparation of ZIF-8@CelF and ZIF-67@CelF

Typically, 2 g of CelF (oven dry basis) and 4 mmol of zinc nitrate hexahydrate were put into a 500 mL three-necked bottle that was placed in a water bath at a temperature of 25 °C, and then 150 mL of deionized water was added. After stirring for 3 h, 50 mL of aqueous solution, containing 240 mmol of 2-MI, was slowly dropped into the above system. After 6 h of reaction, the product was filtered and washed with 10 L of deionized water to obtain the ZIF-8@CelF composite.

The preparation conditions of ZIF-67@CelF were similar to those of ZIF-8@CelF. Only 4 mmol of cobalt nitrate hexahydrate was used to replace zinc nitrate hexahydrate.

#### 2.3.2. Preparation of HKUST-1@CelF

Typically, 2 g of CelF (oven dry basis), 6 mmol of copper acetate and 180 mL of deionized water were put into a 500 mL three-necked bottle that was placed in a water bath at a temperature of 25 °C, and the mixture was stirred for 3 h. Then, 20 mL of absolute ethanol solution, containing 4 mmol of H_3_BTC, was added dropwise to the above system and continuously stirred for 3 h. The product was filtered and washed with 10 L of deionized water to obtain HKUST-1@CelF.

#### 2.3.3. Preparation of MIL-100(Fe)@CelF

Typically, 2 g of CelF (oven dry basis) and 5.7 mmol of ferrous chloride tetrahydrate were put into a 500 mL three-necked bottle, then 150 mL of deionized water was added, and the mixture was stirred at 23 °C for 3 h to ensure the sufficient adsorption of Fe^2+^ by CelF. Then, 3.8 mmol of H_3_BTC, dissolved in 50 mL of aqueous solution containing 11.4 mmol of NaOH, was slowly added to the three-necked bottle, and the mixture was constantly stirred for 24 h. The product was filtered and washed with 10 L of deionized water to obtain MIL-100(Fe)@CelF.

### 2.4. Calculation of MOF Deposition Ratio

The as-prepared product was dried in an oven at 105 °C for 6 h. After cooling for 30 min in a dryer, the mass was measured. The deposition ratio (*D*, %) of MOF was calculated according to the following formula:

*D* = (*M*_2_ − *M*_1_)/*M*_0_ × 100% (1)

where *M*_0_ is the original mass of CelF (g), *M*_1_ is the mass of the composite coated PANI or DPANI layer (g), and *M*_2_ is the mass of the composite deposited MOF (g).

### 2.5. Adsorption-Photocatalysis Experiment

In order to compare the CIP removal ability of the composites with different MIL-100(Fe) loading amounts, MIL-100(Fe)@CelF and MIL-100(Fe)@DPANI@CelF samples with 6 loading levels were prepared by changing the FeCl_2_ dose (2.85, 5.7, 8.55, 11.4, 14.25 and 17.10 mmol) at 1.5:1:3 of the constant FeCl_2_:H_3_BTC:NaOH molar ratio. The above products are marked as T1–T6 in the order of FeCl_2_ dose from low to high.

CIP aqueous solution (160 mg L^−1^) was prepared, and 200 mL were taken each time for subsequent testing. Firstly, 0.25 g of sample (DPANI@CelF, MIL-100(Fe)@CelF and MIL-100(Fe)@DPANI@CelF, oven dry basis) was added to the above CIP aqueous solution and stirred at 500 rpm for 60 min at room temperature and in dark condition to reach the adsorption equilibrium. Then, 250 μL of 30% H_2_O_2_ was added and the system was placed under a CEL-PF300L-3A xenon sunlight-simulating lamp (Beijing China Education AuLight Technology Co., Ltd., Beijing, China) for photocatalytic reaction for 2 h. During photocatalysis, the working current of xenon lamp was adjusted to 20.7 A, and the distance between the lamp and the liquid surface was about 10 cm. Samples were taken every 10 min during adsorption and every 20 min during photocatalysis. CIP concentration was measured by a TU-1950 UV-Vis spectrophotometer at 277 nm.

**Figure 1:**
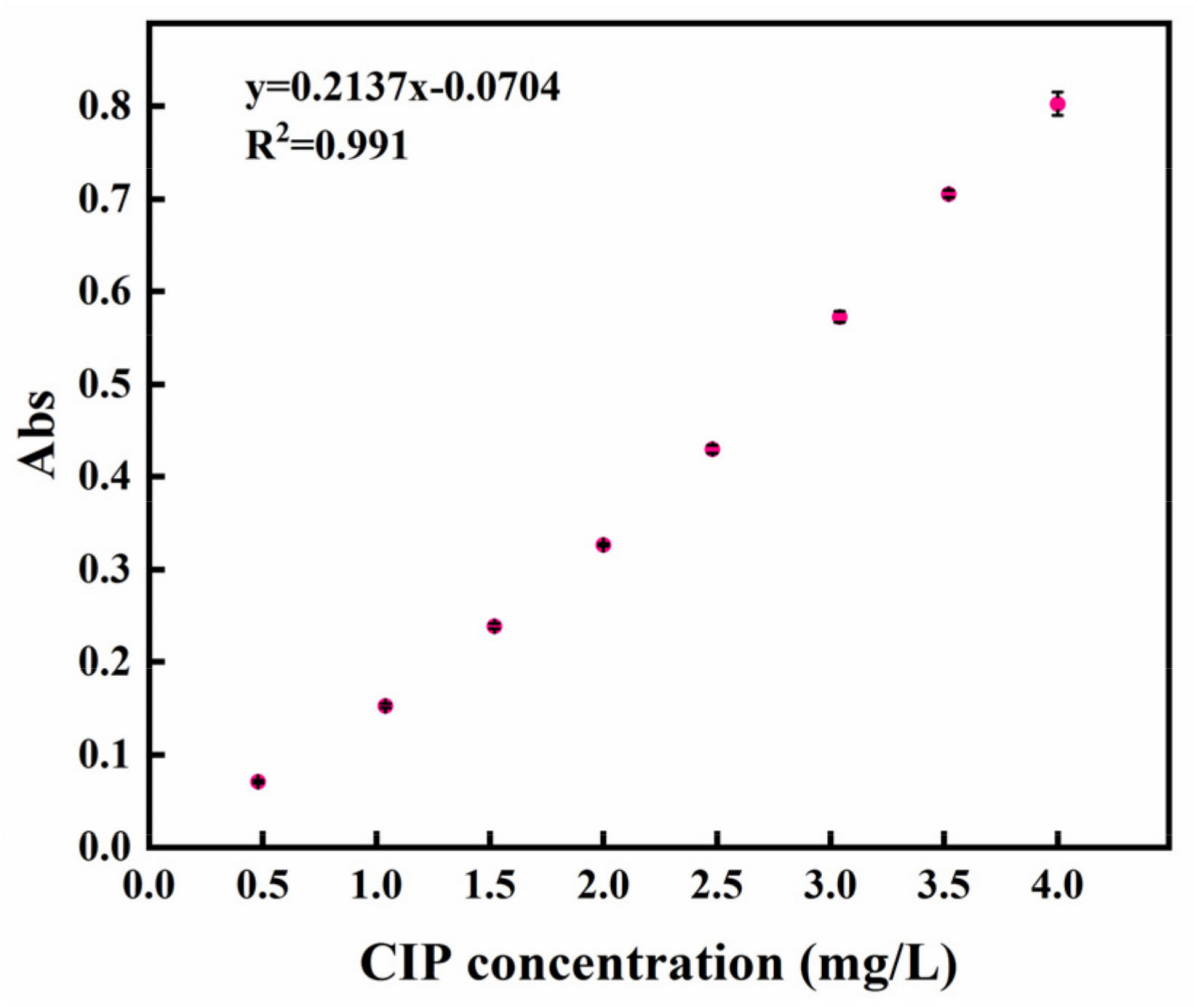
CIP standard working curve.

In order to calculate the removal efficiency of CIP by the composites, the absorbance at 0.48, 1.04, 1.52, 2.00, 2.48, 3.04, 3.52 and 4.00 mg L^−1^ CIP concentrations were measured and the CIP standard working curve was obtained (Figure 1). After linear fitting, the obtained standard working curve equation of CIP was *y* = 0.2137*x* − 0.0704 (*R*^2^ = 0.991), where *y* is the absorbance of CIP at 277 nm and *x* is the mass concentration of CIP (mg L^−1^).

According to the CIP standard curve equation, the CIP concentration corresponding to each sampling stage was calculated, and the removal efficiency (*R*) of CIP by the composite was obtained. The following calculation formula for *R* (%) was used:

*R* = (*C*_0_ − *C*_t_)/*C*_0_ × 100% (2)

where *C*_0_ is the initial mass concentration of CIP (mg L^−1^), and *C*_t_ is the mass concentration of CIP at time *t* (mg L^−1^).

### 2.6. Characterization

The crystalline nature of the samples was analyzed using an X’Pert3 Powder X-ray diffractometer (Panaco, Holland). The ray wavelength was 0.154 nm, the voltage was 40 kV, the current was 40 mA, the scanning range was 5–50°, and the scanning speed was 5° min^−1^. Scanning electron microscopy (Zeiss Supra 55, Germany) was used to analyze the morphology of the samples. The samples containing Fe were coated with gold, and the surface element composition of the samples was analyzed by energy dispersive X-ray spectroscopy (EDS). XPS photoelectron spectroscopy (Thermo Scientific K-Alpha, USA) was used to measure to the element composition and valence information of samples using Al Kα X-ray (1486.6 eV) as the excitation source. FTIR spectra were recorded using a Thermo Scientific Nicolet 10 FTIR spectrometer (USA) in the frequency range of 4000–600 cm^−1^.

## 3. Results and Discussion

### 3.1. Design and Preparation of MOF@DPANI@CelF Composites

**Scheme 1:**
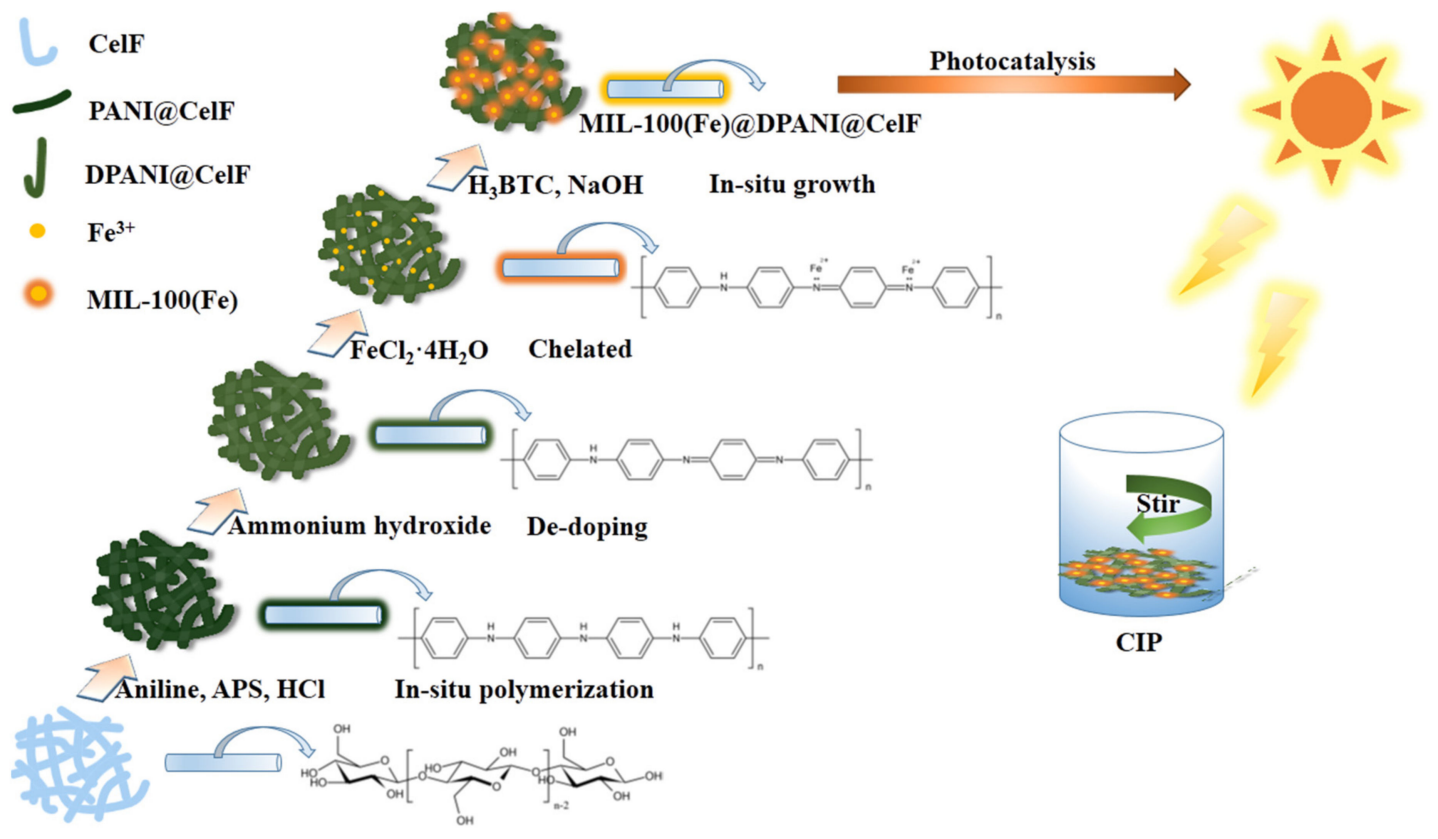
Schematic illustration of the preparation and application of MIL-100(Fe)@DPANI@CelF.

Herein, MIL-100(Fe)@DPANI@CelF composite is taken as an example to briefly illustrate the principle and process of the composite preparation, as shown in Scheme 1. Firstly, a firm PANI layer was formed on the surface of CelF by in-situ polymerization, and then the PANI-mediating layer was de-doped by excessive ammonium hydroxide solution to expose a large number of quinone nitrogen atoms. Next, Fe^2+^ ions were adsorbed on the de-doped PANI with rich binding sites. Subsequently, the deprotonated ligand (BTC^3−^) was added to prompt the in-situ growth and immobilization of MIL-100(Fe) on the DPANI@CelF substrate. Finally, the resulting composite was used in subsequent adsorptive-photocatalytic experiments to remove CIP in the water environment.

### 3.2. Effect of PANI and DPANI as Mediating Layer on Growth of MOFs on CelF

**Figure 2:**
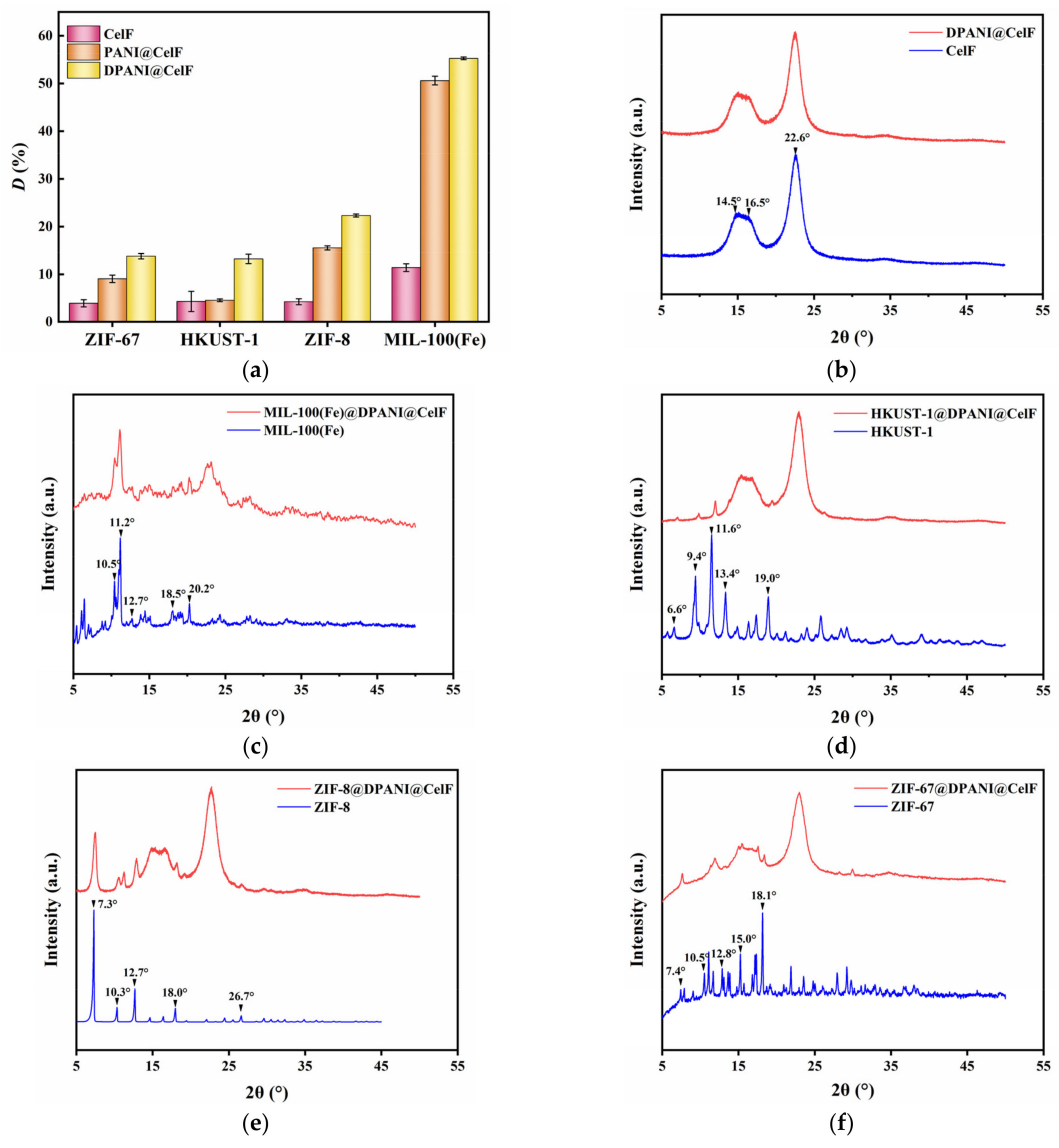
(**a**) Comparison of MOFs’ growth on three CelF substrates; (**b**) XRD patterns of CelF and DPANI@CelF; (**c**–**f**) XRD patterns of MOFs and their composites.

The four MOFs (ZIF-8, ZIF-67, HKUST-1 and MIL-100(Fe)) were in-situ deposited on the three CelF substrates (CelF, PANI@CelF and DPANI@CelF), respectively, and the deposition ratios are shown in Figure 2a. The results show that the existence of the PANI layer significantly improved the in-situ growth and immobilization of the other three MOFs, except for HKUST-1 on CelF. For HKUST-1, no promoting effect of PANI was observed, perhaps due to the micron-rod structure of HKUST-1 [35]. However, the DPANI layer obtained by further de-doping treatment played a positive role in promoting the in-situ growth and immobilization of the four above-mentioned MOFs on CelF. More encouragingly, it was found that the PANI/DPANI layer had an amazing promoting effect on the deposition of MIL-100(Fe) compared with the three other MOFs.

Because of their unique crystalline nature, the formation of MOFs is easily confirmed through the comparison of XRD patterns. As shown in Figure 2b, DPANI@CelF exhibited no significant difference in terms of XRD results as compared with CelF, which shows that the existence of the DPANI layer had no effect on the original structure of CelF. The peaks at 2θ = 10.5°, 11.2°, 12.7°, 18.5°, 19.2° and 20.2° were the characteristic diffraction peaks of MIL-100(Fe). In Figure 2c, it can be seen that the diffraction peaks of MIL-100(Fe)@DPANI@CelF and pure MIL-100(Fe) powder prepared under the same conditions were consistent with those reported in the literature [36], which preliminarily confirms the generation of MIL-100(Fe) on DPANI@CelF. Figure 2d–f demonstrate that HKUST-1, ZIF-8 and ZIF-67 particles were in-situ deposited on DPANI@CelF although some peaks were weak due to the relatively low proportion of MOFs in the composites [35,37,38].

### 3.3. Structure and Morphology of MIL-100(Fe)@DPANI@CelF

**Figure 3:**
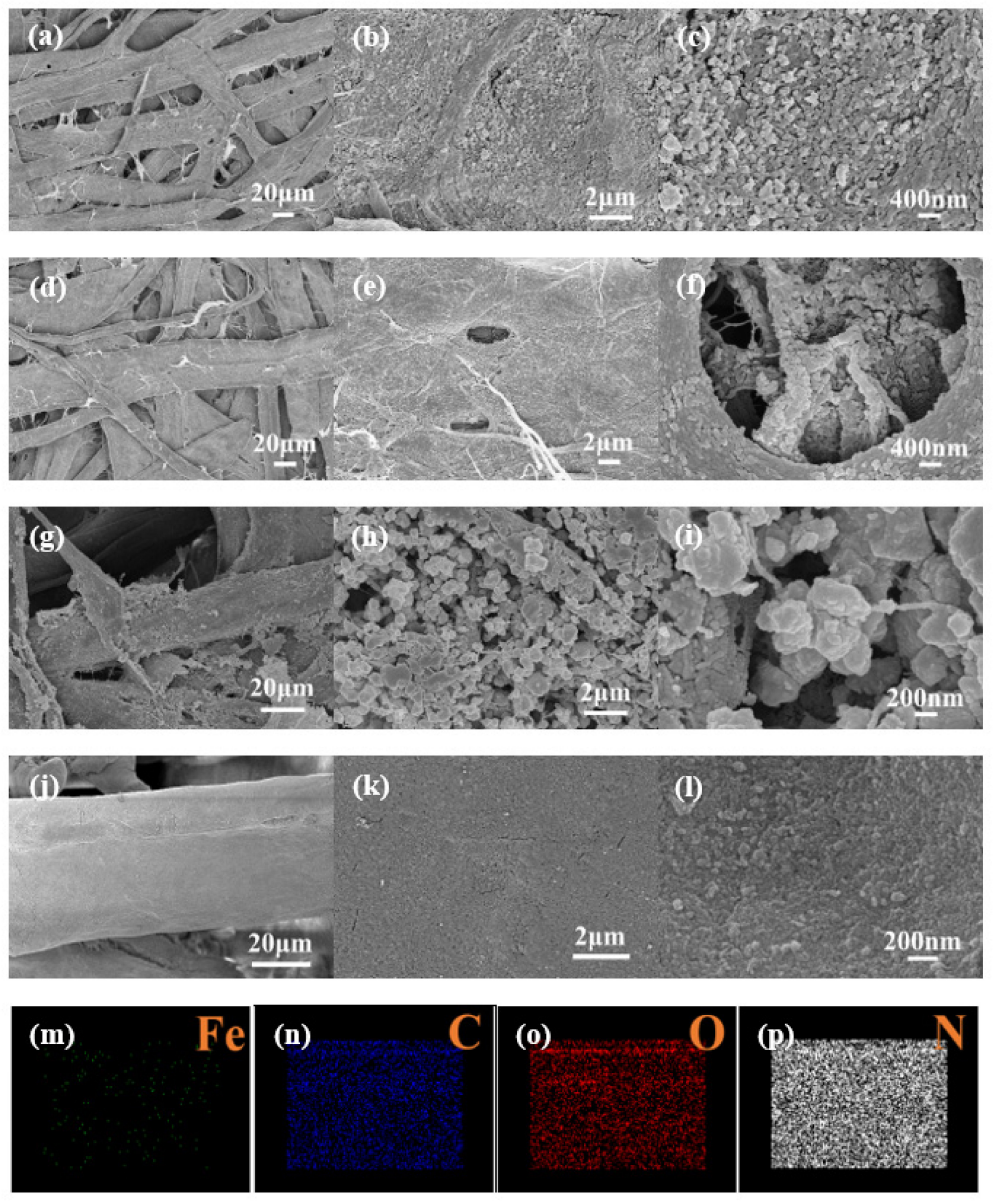
SEM images of PANI@CelF (**a**–**c**), DPANI@CelF (**d**–**f**), MIL-100(Fe)@CelF (**g**–**i**), MIL-100(Fe)@DPANI@CelF (**j**–**l**); EDS images of MIL-100(Fe)@DPANI@CelF (**m**–**p**).

As confirmed by the above results, the loading amount of MIL-100(Fe) on DPANI@CelF was considerable. Next, MIL-100(Fe)@DPANI@CelF, as the main research object, is discussed in detail. Figure 3a–f show the surface morphology of PANI@CelF and DPANI@CelF, respectively, and amorphous PANI/DPANI particles with sizes of about 300 nm, attached to the fiber surface, can be observed. It can also be observed that both the surface and the pits of the fiber were covered with PANI/DPANI particles. The pits were typical of the structure of the fiber surface, i.e., thin areas without secondary walls. Figure 3g–l show the distribution of MIL-100(Fe) on CelF and DPANI@CelF, respectively. It is obvious that the micron-scale irregular MIL-100(Fe) particles, with sizes of about 1 μm, were unevenly distributed on the CelF surface, while the MIL-100(Fe) particles on the DPANI@CelF surface were generally nanoscale (less than 100 nm). This may be because the DPANI@CelF had more nucleation sites, which promoted the in-situ growth and immobilization of more MIL-100(Fe) particles on the DPANI@CelF surface and limited the growth of the larger crystals, to a certain extent, due to the space-charge-limited effect. Nanoscale MIL-100(Fe) particles had much larger specific surface areas, which also positively promoted the follow-up adsorption-photocatalysis. As depicted in Figure 3m–p, the element maps of MIL-100(Fe)@DPANI@CelF show the uniform distribution of Fe, C, O and N elements on the surface of the composite, which further indicates the successful synthesis of MIL-100(Fe) on the surface of DPANI@CelF.

**Figure 4:**
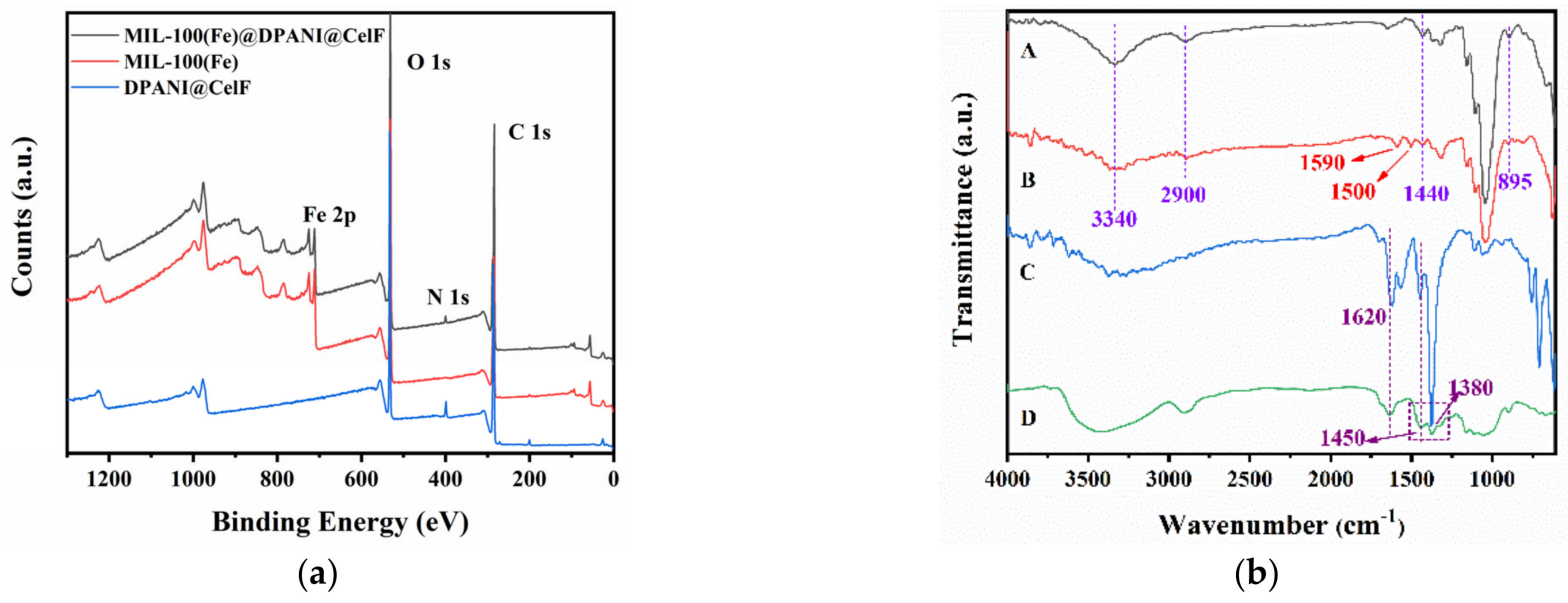
(**a**) XPS spectra of DPANI@CelF, MIL-100(Fe) and MIL-100(Fe)@DPANI@CelF; (**b**) FTIR spectra of CelF (A), DPANI@CelF (B), MIL-100(Fe)@DPANI@CelF (C) and MIL-100(Fe) (D).

The elemental composition and valence states of three samples (MIL-100(Fe), MIL-100(Fe)@DPANI@CelF and DPANI@CelF) were measured by XPS, and the results are shown in Figure 4a. The peaks of C 1s (285.05 eV), O 1s (532.08 eV), and Fe 2p (725.08 eV, 711.08 eV) can be observed in the XPS spectrum of MIL-100(Fe), and the peaks of C 1s, O 1s and N 1s (399.08 eV) are visible in the XPS spectrum of DPANI@CelF, and all of the peaks (C 1s, O 1s, N 1s and Fe 2p) are present in the XPS spectrum of MIL-100(Fe)@DPANI@CelF sample. The C/Fe atomic ratio of the prepared MIL-100(Fe) was 10.9:1, which implies that each Fe^3+^ ion was coordinated with only about one H_3_BTC ligand in the prepared MIL-100(Fe).

The FTIR spectra of CelF, DPANI@CelF, MIL-100(Fe) and MIL-100(Fe)@DPANI@CelF were obtained to analyze their chemical structure, and the results are shown in Figure 4b. For CelF, the vibrational absorption peak of β-glycosidic bond appeared at 895 cm^−1^, the peaks at 1440 cm^−1^ and 2900 cm^−1^ corresponded to the stretching vibrations of H−C−H and C−H, and the absorption peak at 3340 cm^−1^ was caused by the −OH vibration (Spectrum A). For DPANI@CelF, the peaks at 1500 cm^−1^ and 1590 cm^−1^ correspond to the stretching vibration of benzoquinone structure in PANI [28], and these appeared in addition to the characteristic peaks of CelF, which also indicate the successful assembly of the PANI coating on CelF (Spectrum B). Spectrum D shows the characteristic peaks of MIL-100(Fe). The peaks at 1380 cm^−1^ and 1450 cm^−1^ correspond to the symmetric and asymmetric vibration of the –O–C–O–group, respectively, and the vibration peak at 1620 cm^−1^ can be attributed to the interaction between iron ion and −COOH [39]. The characteristic peaks of MIL-100(Fe) also appeared in Spectrum C, indicating that the MIL-100(Fe) particles were grown on DPANI@CelF, which is consistent with the results obtained via XRD, SEM and XPS. However, the characteristic peaks of CelF and DPANI were essentially not displayed in Spectrum C, because they were masked by a large amount of MIL-100(Fe) loaded onto DPANI@CelF.

### 3.4. Removal of CIP in Water by MIL-100(Fe)-Loaded CelF Composites

**Figure 5:**
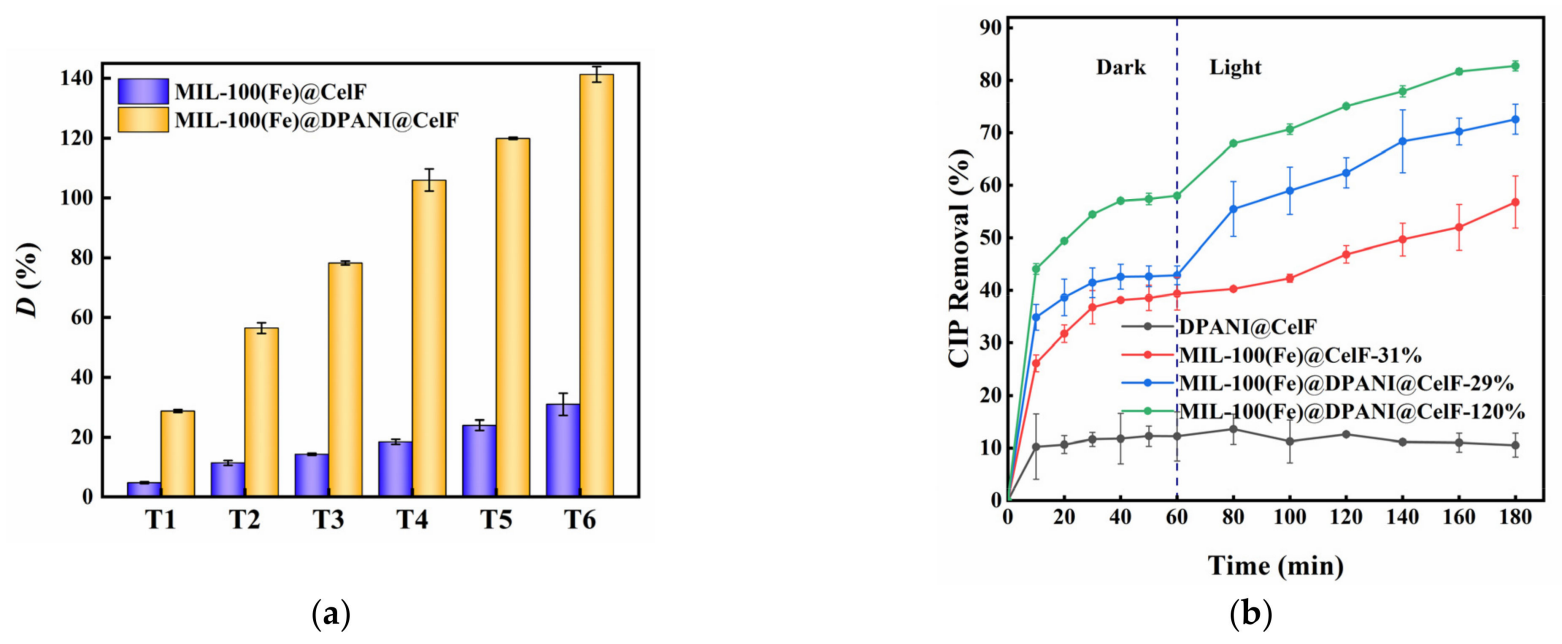
(**a**) Comparison of MIL-100(Fe) deposition on CelF and DPANI@CelF; (**b**) comparison of CIP removal by CelF composites via adsorption-photocatalysis (28%, 31% and 120% refer to the deposition ratios of MIL-100(Fe), 0–60 min for adsorption and 60–180 min for photocatalysis).

Figure 5a shows the MIL-100(Fe) deposition ratios of MIL-100(Fe)@CelF and MIL-100(Fe)@DPANI@CelF prepared at different FeCl_2_ doses. The MIL-100(Fe) deposition ratio almost linearly increased with the increase in the initial FeCl_2_ dosage, and the MIL-100(Fe) deposition ratios of MIL-100(Fe)@DPANI@CelF were surprisingly higher than those of MIL-100(Fe)@CelF in all cases. Figure 5b shows the CIP removal ability of several composites. It was found that DPANI@CelF only had a small amount of adsorption for CIP and had no photocatalytic activity. H_2_O_2_ alone had little effect on CIP removal under experimental light. As the donor of hydroxyl radicals (·OH) in Fenton-like reaction systems, H_2_O_2_ itself does not have photocatalytic activity. Compared with the MIL-100(Fe)@CelF-31%, which had a similar MIL-100(Fe) deposition ratio, the MIL-100(Fe)@DPANI@CelF-29% showed higher adsorption capacity and better photocatalytic performance, highlighting the synergistic promotion of DPANI for photocatalytic reactions. The MIL-100(Fe)@DPANI@CelF-120%, with a higher MIL-100(Fe) deposition ratio, showed higher adsorption capacity and better photocatalytic performance than the MIL-100(Fe)@DPANI@CelF-29%, indicating that the photocatalytic activity of the composite had a certain dependence on the amount of MIL-100(Fe). The existence of the MIL-100(Fe)@DPANI heterojunction improved the absorption and utilization of light and promoted the separation of electrons and holes, thereby enhancing the photocatalytic ability and efficiency. Therefore, DPANI not only greatly promoted the growth of MIL-100(Fe), but also significantly enhanced the photocatalytic activity of the composite. After 3 h adsorption-photocatalysis, the removal ratio of CIP approached 82.78%, and the removal capacity of CIP was as high as 105.96 mg g^−1^.

## 4. Conclusions

In this work, a universal and efficient platform for in-situ growth and immobilization of MOFs on CelF was proposed. It was found that PANI (especially DPANI) could be used as a mediating layer to promote the in-situ growth and immobilization of some MOFs (particularly MIL-100(Fe)) on CelF. The DPANI layer not only significantly promoted the in-situ growth of MIL-100(Fe) on CelF, but also markedly improved the nanocrystallization of MIL-100(Fe). In addition, MIL-100(Fe)/DPANI heterojunction enhanced the photocatalytic ability of the MIL-100(Fe)@DPANI@CelF composite. A small amount of MIL-100(Fe)@DPANI@CelF composite was used to effectively remove CIP in water; the removal ratio of CIP approached 82.78% and the removal capacity of CIP was as high as 105.96 mg g^−1^ after 3 h of adsorption-photocatalysis. This research realized the efficient combination and utilization of CelF, MOFs and PANI through a green and simple method, and provided new insights for the research and development of MOFs@CelF composites.

## Data Availability

The authors confirm that the data supporting the findings of this study are available within the article.
